# Unilateral Biportal Endoscopic Resection of Thoracic Hemivertebra in a Child: A Case Report Highlighting Technical Feasibility and the Critical Importance of Stabilization

**DOI:** 10.1002/ccr3.71596

**Published:** 2025-12-04

**Authors:** Peng Zhang, Chengyun Wang, Zijie Sun, Zhangyong Tan, Kefeng Xu, Genyang Jin, Qi Liu

**Affiliations:** ^1^ Department of Orthopedics The 904th Hospital of Joint Logistic Support Force of PLA Wuxi China; ^2^ Department of Pediatric Orthopedics The First Affiliated Hospital of Xiamen University Xiamen China

**Keywords:** congenital scoliosis, hemivertebra, minimally invasive, nonunion, pediatric, percutaneous pedicle screw, unilateral biportal endoscopy

## Abstract

Unilateral biportal endoscopy (UBE) is technically feasible for complete thoracic hemivertebra resection in young children. However, combining it with unilateral short‐segment percutaneous fixation proved biomechanically insufficient, leading to instrumentation failure and deformity progression. Mechanical stability, potentially requiring bilateral fixation, remains paramount in pediatric deformity correction, even when pursuing minimal invasiveness.

## 1 Background

Hemivertebra (HV) is a common cause of congenital scoliosis (CS), resulting from a failure of vertebral formation [1]. It often leads to progressive, asymmetric spinal growth that is resistant to conservative management, typically necessitating early surgical intervention [2, 3]. Posterior‐only HV resection with short‐segment bilateral pedicle screw fixation is the current standard of care, demonstrating correction rates of 54%–77% in open approaches [4, 5, 6, 7, 8].

Unilateral biportal endoscopy (UBE), a minimally invasive technique well‐established for degenerative spinal conditions [9, 10, 11, 12, 13, 14], offers potential advantages of reduced tissue trauma and enhanced visualization. However, its application in pediatric HV resection remains unreported. This report describes the first case of complete UBE thoracic HV resection (Schwab Grade 5 osteotomy) in a child. We detail the technical execution and critically analyze the subsequent failure of unilateral instrumentation, underscoring the critical balance between minimally invasive goals and the mechanical demands of deformity correction.

## 2 Case Presentation

**FIGURE 1:**
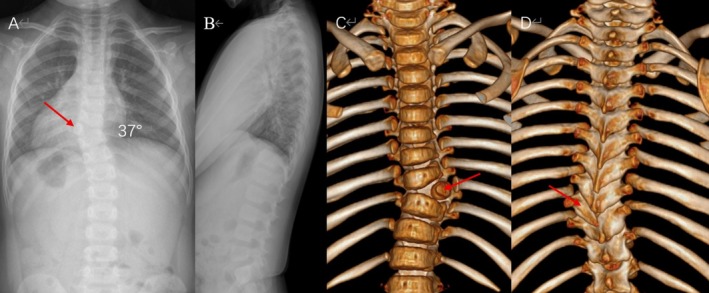
Preoperative imaging. (A) Anteroposterior (AP) radiograph showing thoracic scoliosis (Cobb angle: 37°); (B) lateral radiograph showing physical sagittal alignment; (C) anterior view of 3D CT reconstruction; (D) posterior view of 3D CT reconstruction. Arrows denote the T9 HV in all panels.

A 5‐year‐old male (height: 112 cm, weight: 19 kg) presented with progressive thoracic scoliosis. Radiographic evaluation confirmed a fully segmented T9 HV with a Cobb angle of 37° (Risser sign 0) and physiological sagittal alignment (Figure 1). The anomaly was identified neonatally and monitored annually, with the Cobb angle progressing from 15° at age 1 to 37° at presentation. Neurological examination was intact, and magnetic resonance imaging (MRI) revealed no spinal cord compression.

### 2.1 Primary Surgical Intervention

**FIGURE 2:**
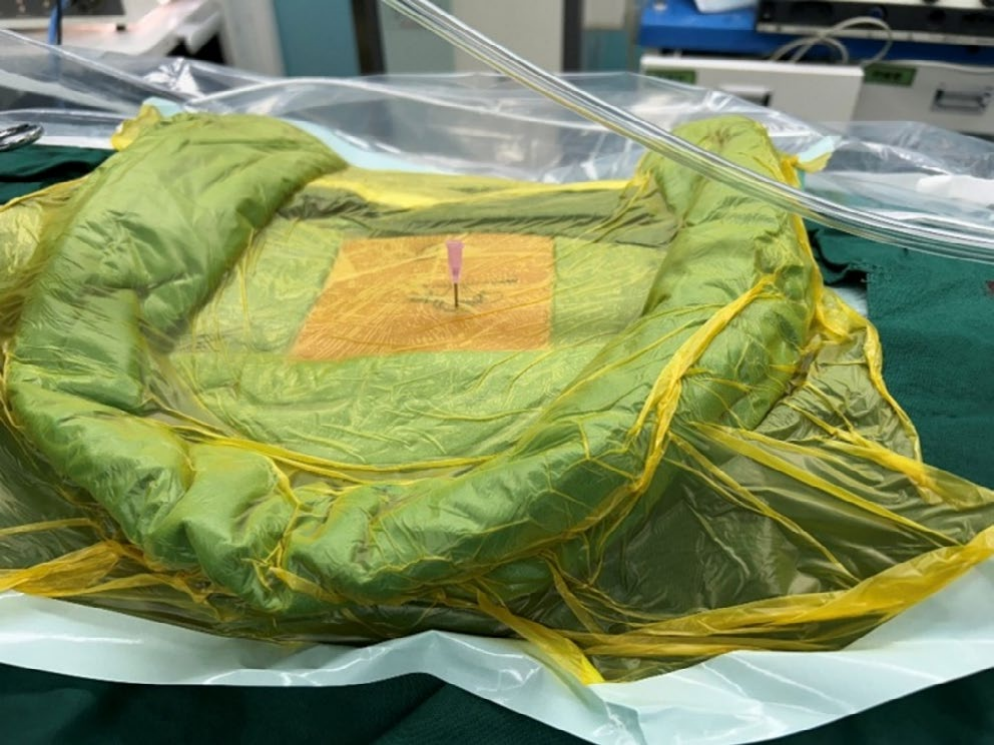
A percutaneous needle was inserted into the left T9 HV pedicle for endoscopic guidance, which was confirmed by intraoperative fluoroscopy.

**FIGURE 3:**
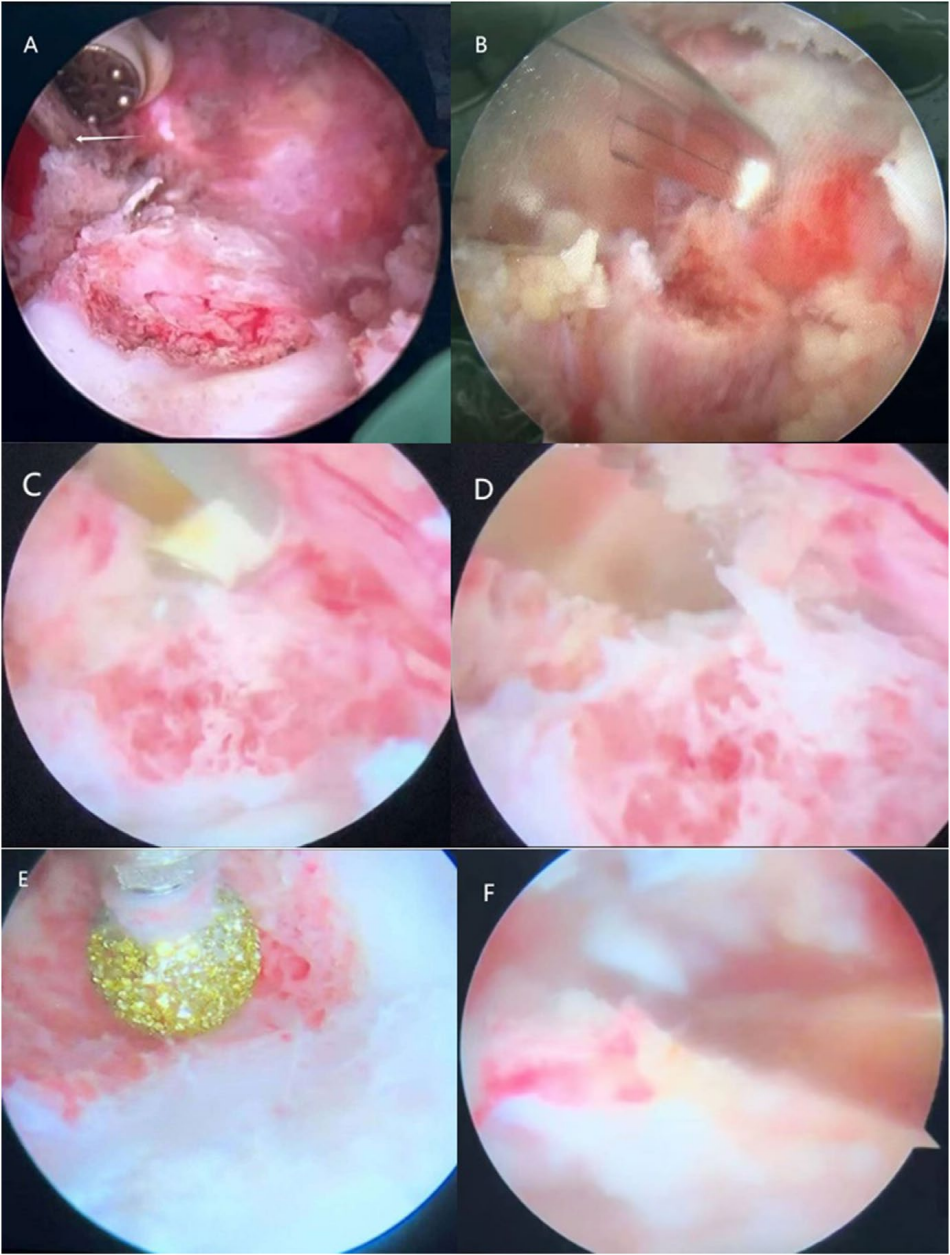
Intraoperative sequence of UBE T9 HV resection. (A) Establishment of endoscopic working space around localization needle (arrow). (B) Resection of rib head and tubercle to expand operative field. (C) Radiofrequency coagulation of pedicular wall vasculature. (D) Osteotomy using ultrasonic bone scalpel. (E) High‐speed drill removal of residual anterior vertebral wall. (F) Complete discectomy under direct visualization.

**FIGURE 4:**
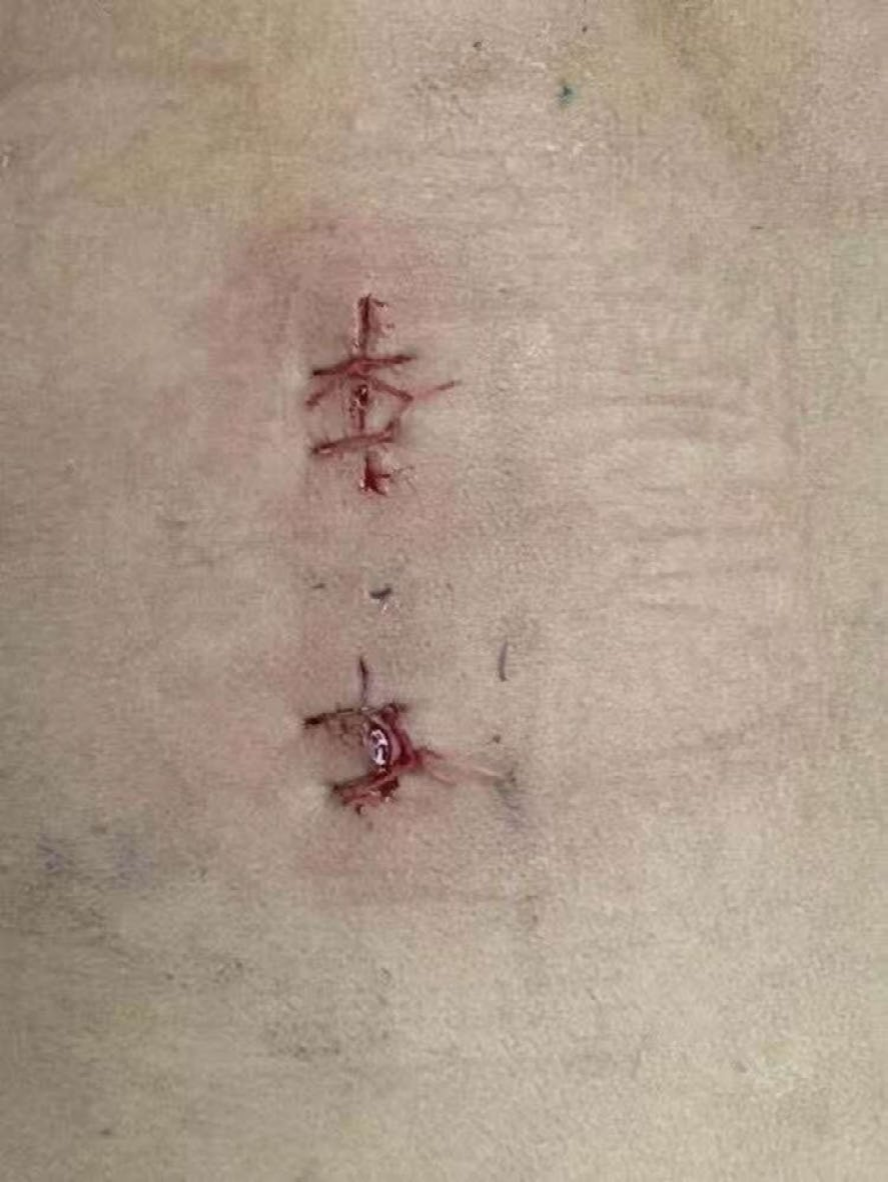
Postoperative view of skin incision.

Under general anesthesia with somatosensory‐ and motor‐evoked potential (SEP/MEP) monitoring, the patient was positioned prone. Intraoperative fluoroscopy localized the HV, and a percutaneous needle was inserted into its pedicle for guidance (Figure 2). Endoscopic viewing and working portals were established proximal and distal to the needle. Soft tissues were coagulated using radiofrequency. The posterior elements of the HV (lamina, facets, transverse process, rib head, and tubercle) were resected using an ultrasonic bone scalpel and Kerrison punch. The pedicle and nerve root were exposed, and the pedicle and vertebral body were resected incrementally; residual components were removed with a high‐speed drill. Adjacent intervertebral disks and endplate cartilage were excised to bleeding bone surfaces (Figure 3). Partial contralateral facetectomy was performed for concave release. Neural elements and pleura were protected under endoscopic vision. Hemostasis was achieved with tranexamic acid and radiofrequency coagulation. The resultant defect was packed with autologous bone chips harvested during the procedure. The smallest available percutaneous pedicle screws (Wego Ortho Premier 5.5 MIS; Ø5 mm, length 30 mm) were placed unilaterally in T8 and T10 via the portal incisions. A pre‐contoured rod was inserted, and gradual compression was applied to close the osteotomy site. Incisions were closed without drainage (Figure 4).

### 2.2 Outcomes and Complications

**FIGURE 5:**
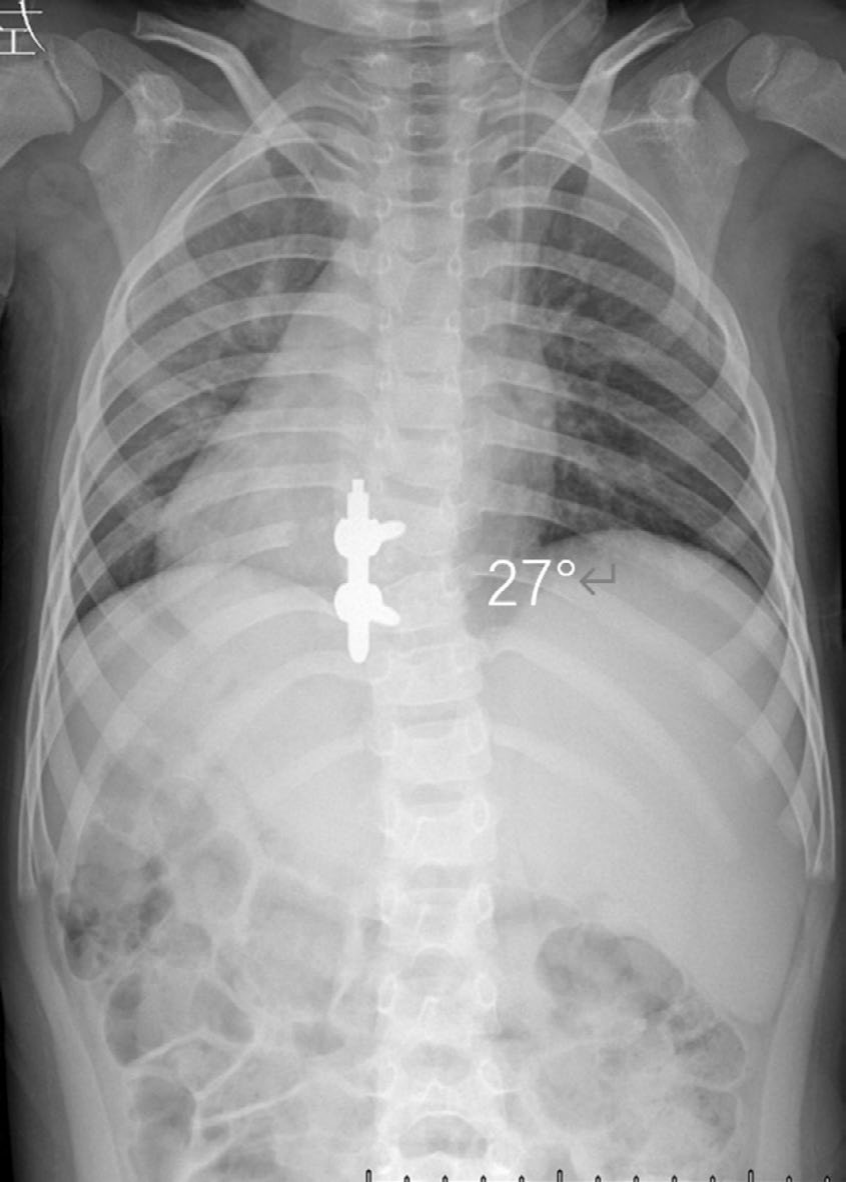
AP radiograph on postoperative Day 1. Initial correction to 27° Cobb angle achieved with unilateral percutaneous pedicle screw fixation (T8–T10; adult‐sized screws: Ø5.0 mm).

The surgical duration was 230 min with minimal blood loss (no transfusion required; hemoglobin remained > 100 g/L). Immediate postoperative radiographs showed a 27% correction of the scoliosis (from 37° to 27°; Figure 5). Neurological status was intact postoperatively. The patient was discharged on postoperative day 10 with a brace prescribed for 6 months.

**FIGURE 6:**
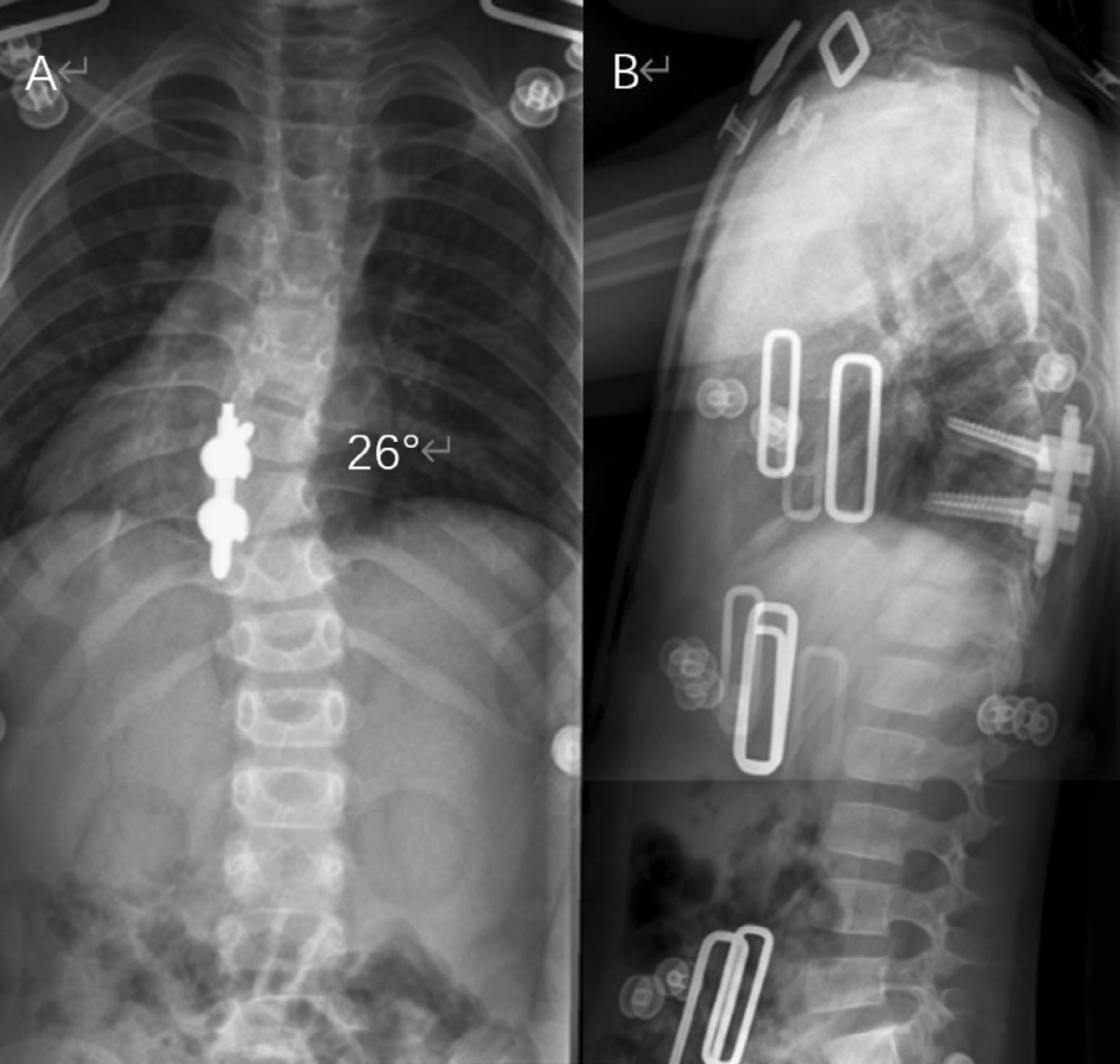
Postoperative 3‐month follow‐up radiographs demonstrating stable alignment prior to fixation failure: (A) AP view showing maintained scoliosis correction (Cobb angle: 26°); (B) lateral view confirming intact sagittal profile.

**FIGURE 7:**
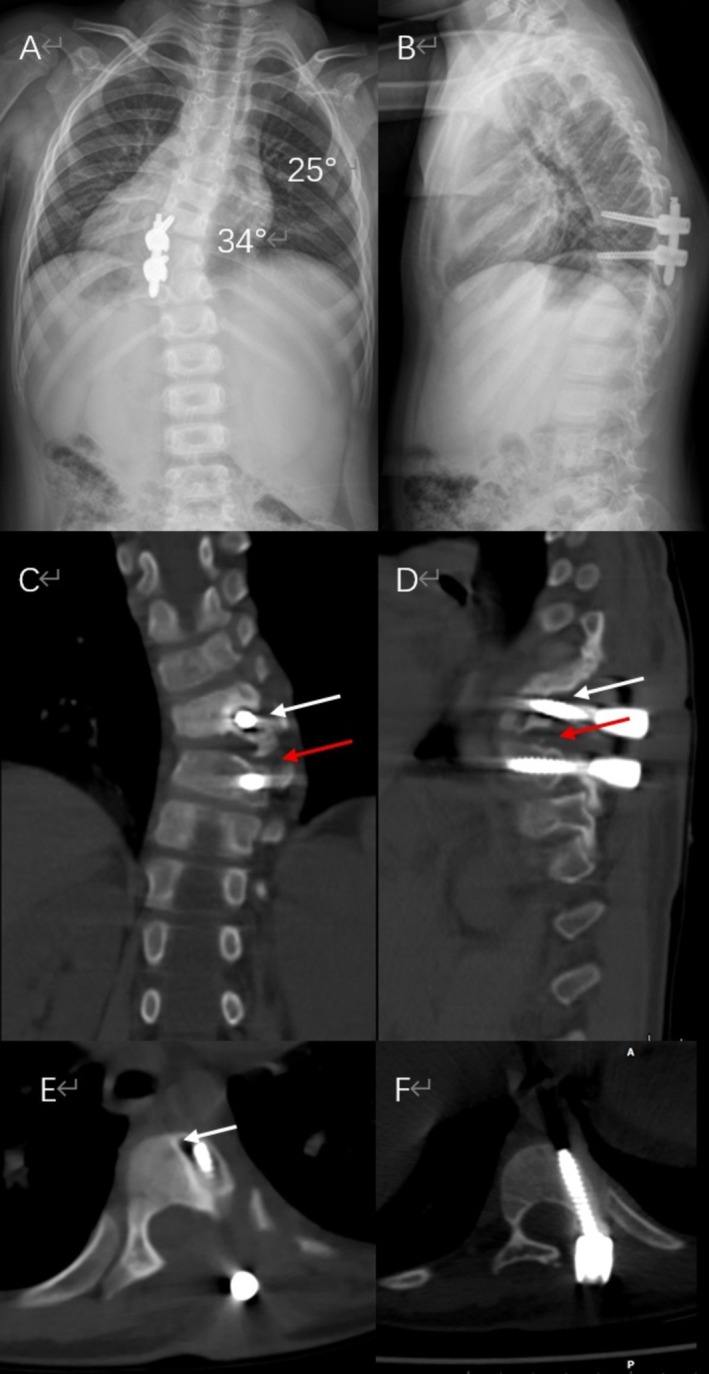
Imaging at 11‐month follow‐up showing instrumentation failure. (A) AP radiograph showing scoliosis progression to 34°. (B) Lateral radiograph revealing kyphosis progression to 25°. (C, D) Coronal and sagittal CT reconstructions indicating nonunion between the graft and T10 vertebral body (red arrow) and circumferential radiolucency (“halo sign”) around the left T8 pedicle screw (white arrow), diagnostic of loosening. (E) Axial CT slice at T8 level highlighting halo sign (white arrow) consistent with screw loosening. (F) Axial CT slice at T10 level demonstrating preserved screw–bone interface without evidence of loosening.

Three‐month follow‐up radiographs indicated stable alignment (Cobb angle 26°; Figure 6). However, at the 11‐month follow‐up, the scoliosis had progressed to 34°, and kyphosis of 25° was noted, although neurological function remained intact. Computed tomography (CT) revealed loosening of the T8 pedicle screw, evidenced by a circumferential radiolucency (halo sign), and nonunion between the grafted bone and T10 vertebral body, creating a functional iatrogenic pseudoarthrosis (Figure 7).

### 2.3 Revision Surgery

Revision surgery was performed via an open posterior approach. The failed implant and grafted bone were removed. The bony surfaces at T8 and T10 were meticulously decorticated. Fresh bone graft was implanted, and bilateral pedicle screws were placed from T7 to T11. Two pre‐curved rods were inserted, and further correction was achieved through convex compression and concave distraction. The wound was closed with a drain after irrigation.

**FIGURE 8:**
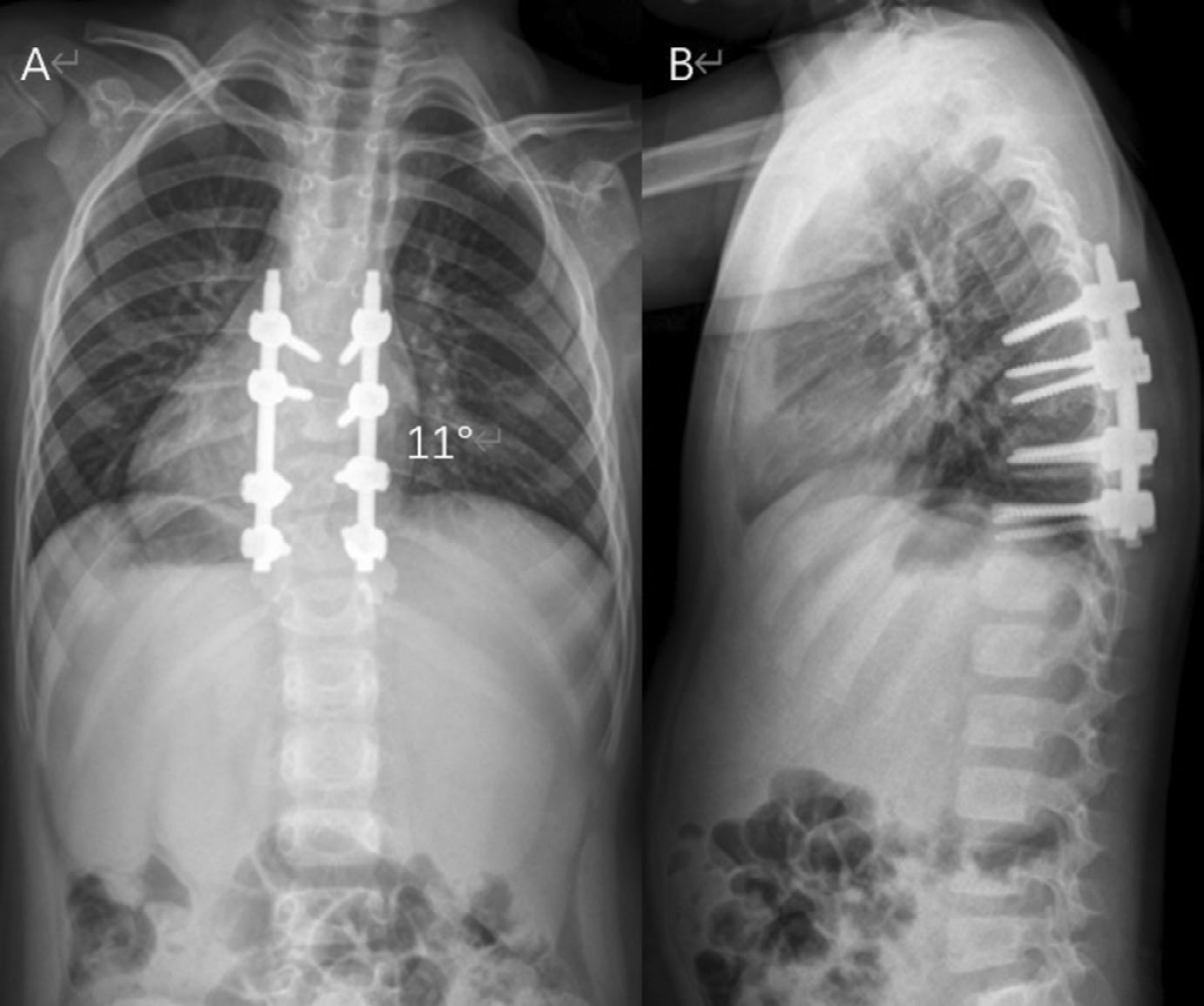
Post‐revision standing radiographs demonstrating 68% scoliosis correction: (A) AP view (Cobb angle: 11°); (B) lateral view. *Note:* Bilateral T7–T11 pedicle screw fixation with restored sagittal alignment.

This 180‐min procedure achieved a 68% correction (from 34° to 11°; Figure 8) with an estimated blood loss of 100 mL. Neurological function was preserved. The patient was discharged on Day 10, again in a brace for 6 months.

## 3 Discussion

This case report demonstrates the technical feasibility of performing a complete thoracic HV resection (equivalent to a Schwab Grade 5 osteotomy or VCR) using the UBE technique in a young child. The endoscopic approach provided magnified visualization, facilitating precise bone removal and neural protection while minimizing muscle trauma. However, the combination of this minimally invasive resection with unilateral short‐segment percutaneous fixation proved biomechanically inadequate, leading to early instrumentation failure (screw loosening), pseudoarthrosis, and deformity progression.

UBE‐specific complications also warrant consideration. While not encountered in this case, the UBE approach carries inherent risks distinct from open surgery. Dural tears are reported in 0.9%–13.2% of UBE procedures [15]. Of particular relevance in the thoracic spine is the risk of iatrogenic parietal pleural rupture, which could lead to massive fluid infusion into the pleural cavity and subsequent cardiopulmonary compromise [16, 17, 18]. Meticulous technique, including partial preservation of pleura‐adherent cortical bone, is recommended for mitigation. These risks underscore the necessity for comprehensive anatomical knowledge and technical mastery when applying UBE to complex spinal deformities.

The failure highlights a critical lesson: The pursuit of minimal invasiveness must not compromise the mechanical stability required for deformity correction, particularly in the pediatric spine undergoing a significant corrective osteotomy. Unilateral short‐segment fixation, while potentially sufficient in some open series [4, 19, 20], was unable to withstand the asymmetric forces in this setting. This was likely due to a combination of reduced load‐sharing capacity, the challenge of achieving optimal screw purchase with percutaneous techniques, and critically, the use of adult‐sized screws (Ø5.0 mm) that were suboptimal for the small pediatric pedicles and prevented adequate bone–screw interface strength. The subsequent successful salvage with open bilateral long‐segment fixation underscores the paramount importance of construct stability.

We hypothesize that the unilateral construct's inability to control the spine, particularly across the resection site, led to micromotion, preventing fusion at T10 and ultimately causing fatigue failure at the T8 screw–bone interface. This case suggests that if UBE resection is employed, it should be coupled with a stabilization strategy proven to provide robust mechanical stability, likely necessitating bilateral screw fixation, potentially with a longer construct, even if placed percutaneously.

From the patient's perspective, this case represented a significant physical and emotional journey. The patient and their family navigated an emotional journey from the initial hope of a fully minimally invasive procedure and the ordeal of long‐term bracing, through the disappointment of correction failure and curve progression, to the ultimate relief of achieving stable alignment and functional improvement after the revision surgery.

## 4 Conclusion

UBE technique is a viable and minimally invasive option for performing complete thoracic HV resection in children. However, this case illustrates that the mechanical demands of subsequent stabilization are immense and cannot be overlooked. Unilateral short‐segment fixation following a major resection like this appears insufficient. Future applications of minimally invasive techniques for congenital deformity correction must prioritize biomechanical stability, potentially involving bilateral percutaneous instrumentation, to prevent failure and ensure successful long‐term outcomes.

## Author Contributions

**Peng Zhang:** data curation, investigation, methodology, writing – original draft, writing – review and editing. **Chengyun Wang:** investigation, writing – original draft. **Zijie Sun:** data curation. **Zhangyong Tan:** data curation. **Kefeng Xu:** investigation, resources. **Genyang Jin:** investigation, resources. **Qi Liu:** conceptualization, funding acquisition, methodology, project administration, supervision, writing – review and editing.

## Funding

This study was supported by the Xiamen Medical and Health Guiding Project under grant 3502Z20209268. The funding source had no involvement in the study design, data collection, analysis, interpretation, writing, or decision to submit this article for publication.

## Ethics Statement

This study was reviewed and approved by the Ethics Committee of The First Affiliated Hospital of Xiamen University. All procedures performed were in accordance with the ethical standards of the institutional and national research committee and with the 1964 Helsinki Declaration and its later amendments or comparable ethical standards.

## Consent

Written informed consent was obtained from the patient's legal guardians for the publication of this case report and any accompanying images.

## Conflicts of Interest

The authors declare no conflicts of interest.

## Data Availability

The data that support the findings of this study are available within the article.
